# Chewable Tablets for Precise Unit Dosing of Animals

**DOI:** 10.1007/s12247-025-10312-0

**Published:** 2025-12-29

**Authors:** Reese E. Landes, Pradeep Valekar, Muslim Abbas, Kieran A. DeLoatch, James B. Schreiber, Ira S. Buckner, Rehana K. Leak

**Affiliations:** Duquesne University, Pittsburgh, PA USA

**Keywords:** Tablets, Oral, Peroral, Oral gavage, Preclinical research, Veterinary, Pest control

## Abstract

**Introduction:**

Peroral administration of preclinical drugs is commonly achieved through oral gavage or food formulations. However, oral gavage induces distress and is unsuitable for behavior studies, whereas food mixing and delivery are less precise. Therefore, we manufactured and tested tablets with the appropriate physical properties for precise unit dosing of laboratory mice.

**Methods:**

Various tablet compositions were evaluated for compactability and compressibility and tested for voluntary consumption by CD-1 mice. The most bitter compound known, denatonium benzoate, was added to tablets at escalating doses, and daily consumption by male and female CD-1 mice was measured across four weeks.

**Results:**

Tablet consumption increased with the number of exposures in 5 of 6 mice, suggesting that a habituation period was necessary. A bitterant was then added at escalating doses (3 to 48 micrograms) for four weeks. After one week of habituation to placebo tablets, 9 of 11 mice ate all bitter tablets, consuming 97% of tablet masses within the first hour. No changes in bodyweight were noted.

**Conclusion:**

These findings support the utility of tablets as a method for drug delivery in laboratory and possibly other settings.

**Supplementary Information:**

The online version contains supplementary material available at 10.1007/s12247-025-10312-0.

## Introduction

Drug administration in animals is commonly achieved through oral gavage [1, 2], as ~ 90% of pharmaceutical formulations on the global market intended for human use are delivered by the peroral route [3]. Although the oral gavage method is relatively precise, physical restraint must be used to force the contents of a syringe down the throat, which can activate the hypothalamic-pituitary-adrenal stress axis [1, 2, 4–7]. Thus, oral gavage increases fecal corticosterone metabolites in mice [6]. Although oral gavage is often performed on conscious animals with practiced technique, other complications include aspiration [2], impacted food/bedding bolus [8], esophageal damage or perforation [4, 7], and an untimely death [4, 8]. In addition, dissolution in dimethyl sulfoxide or corn oil may alter the pharmacokinetic and pharmacodynamic properties of drugs intended for gavage, compared to dry formulations. Finally, the distress induced by oral gavage in conscious rodents renders this method problematic during behavior testing.

Drug-mixing in dietary chow, syrups/spreads, or liquids are common alternatives to oral gavage (e.g., see Supplemental TableS1 and Fig.S1). However, some of these methods may be limited by lack of dosing precision, drug breakdown in liquids, and extended animal handling times. For example, palatable treats have been leveraged to deliver drugs in peanut butter [9, 10], gelatin [11–13], honey [14], bread [15, 16], cereal [17, 18], oatmeal mash [19], oat flakes [20], chocolate spreads [21], cookie dough [6], and cookie pieces [22, 23]. Drugs can be difficult to incorporate evenly into spreadable/sticky treats, and the ingested dose may be imprecisely measured, unless time-consuming handfeeding with a syringe or micropipette is employed (e.g., [24, 25]). Drugs can also be added to liquids, but the drug must be soluble and remain stable, and animals tend to spill liquids, making it difficult to quantify dose. Similarly, measurements of ingested dose are imprecise for dietary chow. Chow pellets are friable, leading to a fine dust strewn across the cage floor. If the timing of drug exposure is spread across the light/dark cycle, *ad libitum* drug administration may also limit clinical translatability. Furthermore, social housing conditions confound measurements of food ingestion [26]. These pitfalls can limit the translational value and reproducibility of preclinical drug testing, wasting resources and time.

To circumvent some of the pitfalls of imprecise or laborious drug delivery methods, such as in friable chow and spillable liquids, or the handfeeding of syrupy treats, the objective of this proof-of-concept study was to manufacture chewable tablets as a solid dosage formulation, evaluate compactibility and compressibility, and test voluntary consumption by mice across four weeks. In tablet manufacturing, drugs are homogeneously distributed across all tablets for relatively precise and consistent dosing, but this process has not been widely adopted for animal studies (TableS1, Fig. S1). After developing an appropriate formulation, we tested the hypothesis that CD-1 mice would continue to consume tablets with escalating concentrations of the bitterant denatonium benzoate—even during periods of *ad libitum* access to regular chow. Measurement outcomes included tablet porosity and mechanical strength, the fraction of each tablet consumed per exposure hour, and body weight changes over a four-week period.

## Methods

### Animals

Fourteen male and female mice of the CD-1 stock were purchased from Charles River (Wilmington, MA) or bred in-house. All the mice were initially surplus animals, and no mice were euthanized when the study was terminated. Thus, all animals were retained for reuse in future experiments, in accordance with the three Rs.

**Fig. 1:**
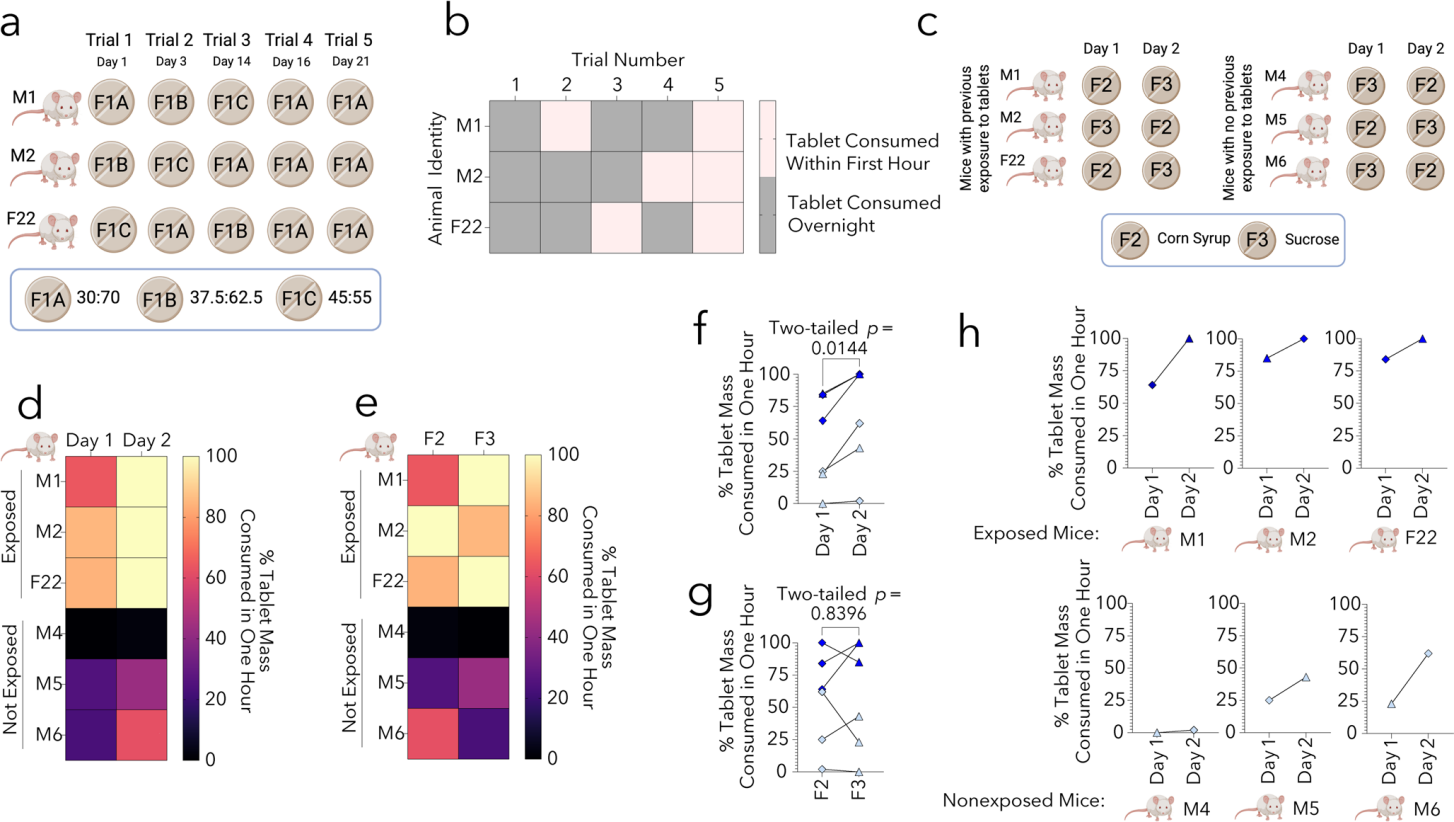
(**a-b**) Two-month-old male and female CD-1 mice were administered tablets of varying compositions five times over a three-week period (schematized in BioRender). The percentage consumed is illustrated as a heatmap (pink boxes represent tablets consumed within one hour and gray boxes represent tablets consumed overnight, within ~ 22 h). Each row represents one mouse, and each square represents one trial. (**c**) Three-month-old male and female mice were given F2 or F3 tablets over two days. F2 and F3 were similar oatmeal formulations, with corn syrup in F2 and sucrose in F3. (**d-h**) The mice from the previous experiment were reused (M1, M2, and F22) as animals with prior tablet exposure, and three additional male mice with no prior exposure to tablets (M4, M5, and M6) were added. The heatmaps in **d** and **e** and scatterplots in **f** and **g** represent the percentage of tablets consumed as a function of time across two consecutive days (**d**,** f**) and as a function of tablet type (**e**,** g**). Mice with previous exposures are depicted in dark blue, and mice with no previous exposure are in light blue. F2 tablets are depicted as diamonds and F3 tablets as triangles (**f-h**). Animals are plotted individually in **h** to illustrate the % consumed across two consecutive days per mouse. Two-tailed paired *t* tests were employed in panels **f-g**

Animals were maintained in a temperature-controlled environment at 20–26 °C with lights on between 0700 and 1900 h. All procedures were conducted in accordance with the *NIH Guide for the Care and Use of Laboratory Animals* and approved by the IACUC (Protocol no. 2300). Experienced animal care personnel with LAT/LATG certifications managed animal maintenance, a board-certified veterinarian supervised animal care (OLAW assurance D16-00562), and biannual inspections were conducted by the IACUC committee. Two-month-old mice were used for the preliminary work in Fig. 1. Although neuroscience research is often performed on young adult rodents of this age, bodyweights have not plateaued at two months of age. Therefore, we transitioned to six-month-old mice for the main experiment that continued for four weeks (Fig. 3). Of those latter eleven mice, three (M4 to M6) had been used in the pilot studies of Fig. 1, four months previously, in accordance with the three Rs.

Mice were socially housed other than during the experimentation period, except in cases of in-cage fighting and wound formation. Before and during experimentation, all animals were permitted *ad libitum* access to Lab Rodent Diet 5001 (Bio-Serv^®^) and UV-disinfected water. When experiments were not ongoing, mice were given treats (Bio-Serv^®^). All mice were housed in polycarbonate caging (28 × 18 × 12 cm) with filter tops and straw-colored Enrich-o’Cobs^®^ bedding (Anderson Labs). Mice were also provided nesting materials, polycarbonate shelters (igloos), and chewing objects (Nylabones or wood blocks). Cages and bedding were not fully changed between tablet administrations to avoid stress [27, 28]. Thus, bedding was changed once a week for singly housed mice and twice a week for socially housed mice, unless excessively soiled.

### Tablet Manufacturing

Our tablet formulations are under patent consideration (U.S. Patent Application Serial No. 18/774,109). Tablets were manufactured from various nutrient blends containing oat bran, wheat germ, yeast, an oil, and a sweetener. These oatmeal blends were combined with the tableting aid silicified microcrystalline cellulose (Prosolve^®^ SMCC 90, JRS Pharma). All solid ingredients were sieved through a 250 μm screen prior to use. The oat bran and wheat germ were co-milled prior to sieving. The ingredients were blended using a lab scale mixer. The composition of the oatmeal blend and amount of tableting aid were altered to determine a functional formulation that was accepted by the animals. A small proportion (< 0.05% w/w) of blue food lake was included to distinguish tablet fragments. To investigate the impact of bitter drugs on the acceptability of the final formulation, the widely used bitterant denatonium benzoate was added to the blend in increasing amounts. Denatonium benzoate anhydrate granules were purchased from Spectrum™ at National Formulary (NF) grade. The compositions of blends compressed into tablets are in Table 1. Tablet materials were purchased from MP Biomedicals ^TM^ (Irvine, California, United States of America), Spectrum ^TM^ (New Brunswick, New Jersey, United States of America), and JRS Pharma (Patterson, New York, United States of America).

**Table 1:** Compositions of tablets offered to animals, expressed as % w/w

Formulation [Manufacturer]	F1A	F1B	F1C	F2	F3	F4
Oat bran [MP Biomedicals™, animal diet grade]	16.5%	20.625%	24.75%	38.5%	38.5%	25.0%
Wheat germ [MP Biomedicals™, animal diet grade]	6.0%	7.5%	9.0%	14.0%	14.0%	10.0%
Yeast [MP Biomedicals™, animal diet grade]	1.5%	1.875%	2.25%	3.5%	3.5%	5.0%
Oil [corn oil: Spectrum™, NF grade or cod liver oil: Spectrum™, USP grade]	3.0% cod liver oil	3.75% cod liver oil	4.5% cod liver oil	7.0% corn oil	7.0% corn oil	5.0% cod liver oil
Sweetener [corn syrup: MP Biomedicals™, animal diet grade, or compressible sugar: JRS Pharma, Sugartab^®^ grade]	3.0% corn syrup	3.75% corn syrup	4.5% corn syrup	7.0% corn syrup	7.0% sucrose	5.0% corn syrup
Tableting aid [JRS Pharma, Prosolve^®^ SMCC 90]	70%	62.5%	55%	30%	30%	50%

Blends were compressed into tablets using a compaction emulator (Presster emulating a 38 station HATA HT-AP38-MSU press operating at 20 rpm). Standard B tooling (round 9.5 mm, concave) was used for all tablets. Our initial study involved three F1 formulations with 15 tablets weighing 405.7 mg ± 6.5. We then tested F2 and F3 tablets (6 tablets of each type), weighing 403.5 ± 11.4 mg. For the final study, we prepared 220 tablets using the F4 formulation, weighing 304.3 ± 4.0 mg. The bitterant was added to the F4 formulation prior to compression, such that the denatonium benzoate content of these tablets varied from zero to 48 µg.

Tablets underwent quality tests, including weight variation, compactibility, compressibility, and friability. The relationship between tablet porosity and compression stress, defined as compressibility, and the relationship between tablet tensile strength and tablet porosity, defined as compactibility, were determined. Porosity was calculated using the tablet outer dimensions measured with a digital caliper following full relaxation, post-ejection, and the true density of the formulations determined using helium pycnometry (Accupyc, Micromeritics). The tensile strength was calculated using the diametral compression force, causing tensile failure, as determined with a hardness tester (Sotax model HT1) and the dimensions of the tablet. Tablet friability was measured using the United States Pharmacopeia (USP) method [29], to evaluate resistance to mechanical stress, prior to administration to the animals.

### Experimental Design

#### Identification of Preferred Tablet Composition

Male and female mice were given five different compositions of placebo tablets. First, we tested formulations F1A-C on three mice (*n* = 2 males, 1 female). Mice were exposed to tablets five times between ~ 10 and 11 AM in their home cages for one hour over three weeks. After this observation period, tablet remnants were left overnight in the cages. All mice received at least one of each tablet type in a randomized order for the first three exposures. For the last two exposures, all animals received F1A tablets (Fig. 1a). The investigator was not blinded to the group assignment in this study.

To test the impact of tablet composition, we tested two variations of formulation F1A. In both F2 and F3, cod liver oil was replaced with corn oil. In addition, corn syrup sweetener was replaced with compressible sucrose powder in F3. In this experiment, the same three mice were used, alongside three new males with no prior exposure to tablets (*n* = 6; 5 males and 1 female). These mice were exposed to both tablet types over two consecutive days in a randomized order (Fig. 1c). The same regimen as above was followed, except that tablet remnants were weighed after the one-hour observation period.

#### Study Design for Denatonium Benzoate Tablets

Male and female CD-1 mice were administered control (F4 placebo tablets) or experimental (F4 denatonium benzoate tablets) tablets, every day during the week (5 days on, 2 days off), for four consecutive weeks (*n* = 11; 7 males and 4 females). After the first week, we performed a dose-escalation study (3 to 48 µg denatonium benzoate). Mice were exposed to tablets from 4 to 5 PM in home cages. After one hour, we sifted manually through bedding and removed all blue tablet remnants for determination of ingested fractions. Thus, pieces of uneaten tablets were readily identified by color contrast with the straw-colored bedding and weighed.

### Statistical Analyses

Statistical tests are specified in figure legends, and each animal is depicted as an individual datapoint in scatterplot graphs. Data on two groups in Fig. 1 were subjected to parametric paired, two-tailed *t* tests after checking Gaussian distributions with the Shapiro-Wilk and Kolmogorov-Smirnov tests. The Wilcoxon matched-pairs signed rank test was also applied to the same data. The repeated measures two-way ANOVA/Dunn nonparametric test by Milton Friedman was used on the non-Gaussian data of Fig. 3. Alpha was set at 0.0500. Data were analyzed with GraphPad Prism (Version 10.4.2) and SPSS (Version 31.0.0.0 (117)).

This study was not powered to evaluate sex differences or the impact of animal age on tablet consumption. Hence, sample sizes for the paired testing of Fig. 1 were not calculated a priori. For the denatonium benzoate study, power was calculated to range from 0.80 to 0.95 for effect sizes between 0.20 and 0.30 (two-tailed α = 0.05; G*Power 3.1.9.7). An *n* of 11 mice was used for the repeated-measures design.

A priori animal exclusion criteria included evidence of sickness (> 20% body weight loss, wound or tumor formation, or an early death), but we did not find evidence of illness. Thus, all animals were included. No outlier test was employed; rather, all data points were included as part of the expected high statistical variation in rodent behavior assays.

## Results

### Habituation Impacts Consumption More Than Tablet Type

First, we evaluated three F1 tablet compositions with varying ratios of oatmeal to SMCC90 (30:70, 37.5:62.5, 45:55; Fig. 1a). No mice consumed the entire tablet during the first one-hour period of Trial 1, but all mice consumed the entire tablet by the following morning (Fig. 1b). In Trial 2, the mouse receiving the 37.5:62.5 tablet ate the entire tablet within 49 min, but the other two consumed it overnight. Similarly, during Trial 3, the mouse receiving the 37.5:62.5 tablets consumed the tablet within the hour, but the other two consumed the tablets overnight. Although the amounts consumed varied after one hour, two of the three mice consumed more of their tablets during Trial 3 than either of the previous trials.

Next, to test the importance of habituation, we completed two more trials (Trials 4–5) on the same cohort, with two additional exposures to 30:70 tablets. In Trial 4, one mouse consumed the entire tablet within four minutes, while the other mice ate the entirety of the tablets overnight. In Trial 5, all mice consumed the entirety of each tablet within the first hour of exposure (range 21–41 min; Fig. 1b). Thus, consumption rate appeared to be associated with the number of exposures, rather than the specific F1 tablet type. This conclusion was further supported by consumption rates of F2 and F3 tablets by the three aforementioned mice with previous tablet exposures (M1, M2, F22) versus new mice *without* previous exposures (M4, M5, M6; Fig. 1c-d). Hence, mice with prior exposure consumed more than mice without previous exposure on both Day 1 and Day 2, and by Day 2, all mice with previous exposure consumed the entire tablet within the first hour (Fig. 1d). In addition, regardless of prior exposures, all mice consumed more of each tablet on Day 2 compared to Day 1 (two tailed *p* = 0.0144 by paired *t* test and *p* = 0.0312 by the Wilcoxon matched-pairs signed rank test; Fig. 1f). As expected, there was no difference in the fraction consumed as a function of tablet type (Fig. 1g).

### 3.2 Optimization of Tablet Compressibility and Compactibility

**Fig. 2:**
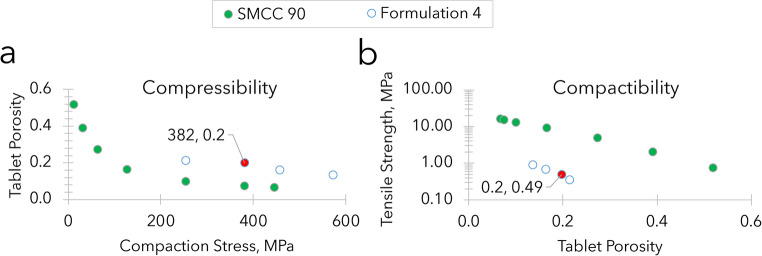
(**a**) Compressibility and (**b**) compactibility of Formulation 4 (open symbols) in comparison to the tableting aid Prosolve SMCC 90 (closed, green symbols), as reference. The properties of the tablet type used in the animal studies are denoted by the filled, red circles. These tablets combined moderate compression stress (< 400 MPa) with tensile strength (~ 0.5 MPa) that balanced chewability against friability. Both the compressibility and compactibility represent tablet properties after viscoelastic relaxation following ejection

Based on our finding that tablet composition played a less significant role than habituation in tablet consumption, we developed composition F4 with improved strength, compactibility, and compressibility (Fig. 2).

### 3.3 A Tablet Bitterant Did Not Affect Tablet Consumption Rates

**Fig. 3:**
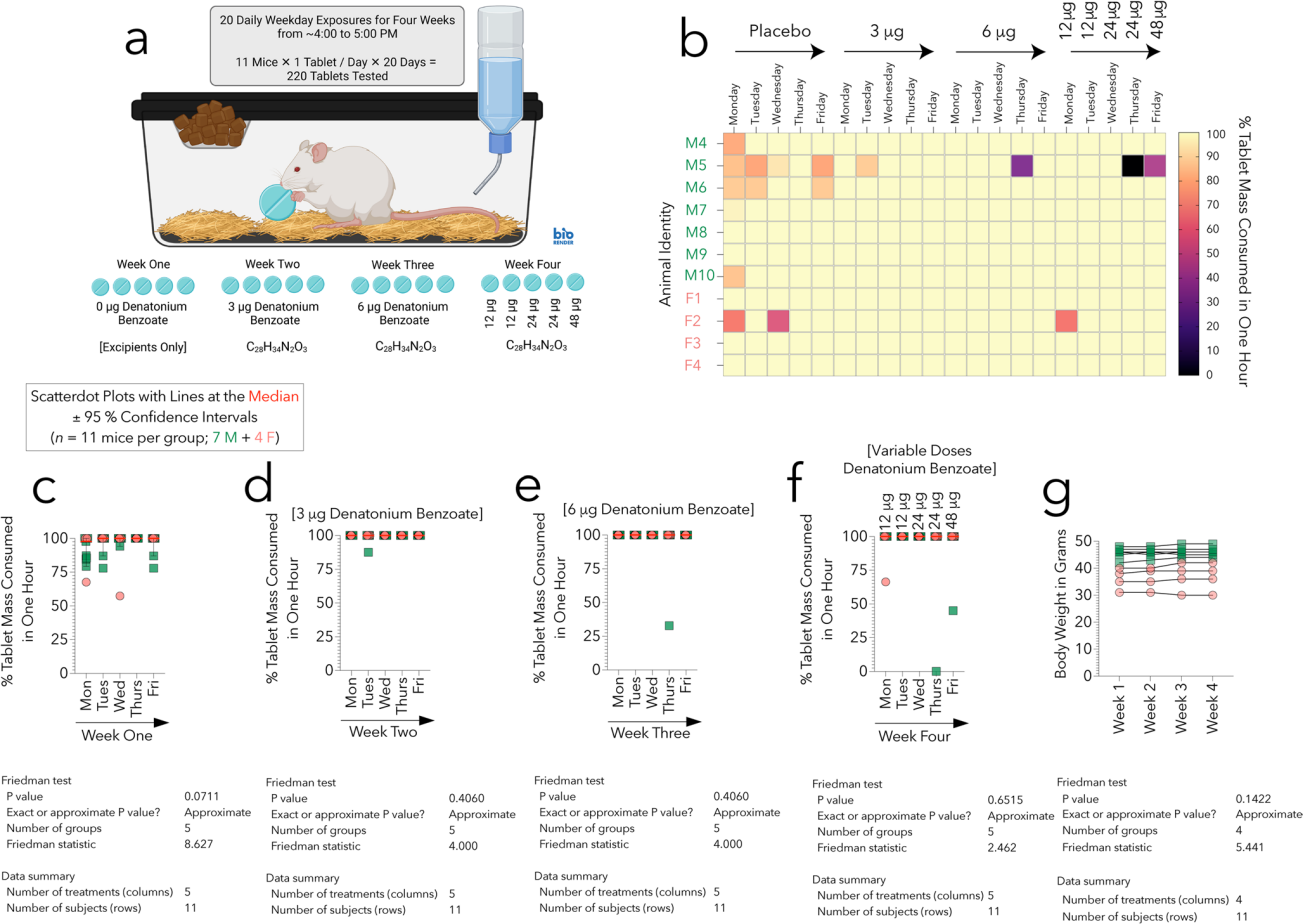
(**a**) Six-month-old male and female mice were given a placebo tablet or a tablet containing denatonium benzoate for four consecutive weeks in their home cages (schematized in BioRender). (**b**) The heatmap shows the % of each tablet consumed per day by each mouse. Each row represents one mouse, and each square represents one day of consumption. Male mice are depicted in green and females in orange. (**c-f**) Scatterplots of % tablet mass consumed are shown for each of four weeks, with lines depicting the median and 95% confidence intervals. (**g**) Bodyweights as a function of time, across the four-week study. The results of the nonparametric, repeated measures two-way Friedman ANOVA/Dunn test are included below corresponding graphs

To test if the addition of a drug would impact consumption rate, we capitalized on the most bitter substance known, denatonium benzoate. Placebo F4 tablets were administered in the first week, and a dose-escalation study was performed for the following three weeks (Fig. 3a). The greatest variation in consumption occurred during the first week of exposure (Fig. 3a-f), as expected based on the pilot work of Fig. 1.

By the start of the second week, nine out of eleven mice (all but M5 and F2) consumed the entire tablet within the first hour, and for the remainder of the study, these nine mice continued to eat the entire tablet within the first hour (15/15 days with entire tablet consumed; Fig. 3b). M5 displayed the greatest variability in consumption and, after the first week, ate the entire tablet 11/15 times. F2 ate the entire tablet 14/15 times after week one (Fig. 3b). Overall, 93% of 220 tablets were consumed within the hour, and after the first week, 97% of 165 tablets were consumed within the hour. No weight changes were observed (Fig. 3g).

## Discussion and Conclusion

Common approaches to pharmaceutical drug-testing in animals include needle injections, oral gavage, or mixing drugs into treats, but each method suffers significant drawbacks. As a precise unit dosing alternative for orally bioavailable drugs, we propose chewable tablets composed of carbohydrates, fats, proteins, and excipients. Application of our method would maintain precise unit dosing in a shorter timeframe compared to *ad libitum* access to chow formulations across the light/dark cycle. With our novel chewable formulation, tablet remnants can be readily collected and weighed for subtractive analyses of the consumed fraction. Tablet consumption increased with exposures irrespective of tablet type, supporting use of a habituation period. After the habituation week, bitter tablets were consumed within the exposure hour by 9/11 mice, every day for three consecutive weeks, with no bodyweight changes. In our vivarium, the CD-1 mice consume, on average, ~ 4.0 g per day (male) or ~ 3.7 g per day (female) at this age (unpublished). Thus, 300 or 400 mg tablets would represent ~ 7.5 to 10% of dietary mass consumed per diem.

Limitations of this study include the following. First, this is a proof-of-concept study on a small number of animals from a single stock, the genetically outbred CD-1 mouse. Second, as mice were exposed to placebo tablets followed by escalating bitterant doses, all in the same sequence, no formal method of mouse randomization could be applied in the main study. A third limitation is that the tablet method would be unsuitable if mice develop an aversion to an experimental drug (e.g., nicotine in [7]) and begin to refuse the tablets. In that event, voluntary administration is unfeasible. Otherwise, we suggest a one-week habituation period to minimize variability in consumption and overcome rodent neophobia [30]. Although the investigator could exclude non-responsive mice or stratify mice *post hoc* according to dose consumed, the exclusion of animals can introduce sources of bias, as it bypasses the animal randomization process. Fourth, our work is limited by the single housing of mice during the testing period, which can induce stress. For future work, we recommend that animals be socially housed for 23 h per day, placed into single housing for one hour (or less) to administer tablets and monitor consumption, followed by reintroduction to familiar cage mates. Mice could also be habituated to this interruption in normal housing conditions for one week prior to drug administration. In addition, we recommend tablet administration at a consistent time of day, to account for circadian rhythms in food intake. The fifth limitation is the need for tablet manufacturing and testing equipment, which may render the methods described herein out of reach for some academic laboratories but may also foster greater collaboration across the pharmaceutical sciences.

In conclusion, the voluntary consumption rates of chewable tablets support their usefulness for drug delivery in scientific research and possibly veterinary and pest control settings. Compared to intermixing drugs with dietary chow and spreadable or liquid treats, tablet manufacturing may minimize dosing imprecision and/or avoid potential problems with drug solubility and degradation.

## Supplementary Information

Below is the link to the electronic supplementary material.

Supplementary Material 1 (PDF 224 KB)

Supplementary Material 2 (XLSX 15.7 KB)

## Data Availability

Data are provided within the manuscript and also available upon emailing RKL and ISB.
