# Supplementary material for: Chewable Tablets for Precise Unit Dosing of Animals
Source: J Pharm Innov. 2025 Dec 29;21(1):81. doi: 10.1007/s12247-025-10312-0 (PMC12748284; doi:10.1007/s12247-025-10312-0)
Supplement: Supplementary file 1 — Supplementary Material 1 (PDF 224 KB) [file 12247_2025_10312_MOESM1_ESM.pdf]

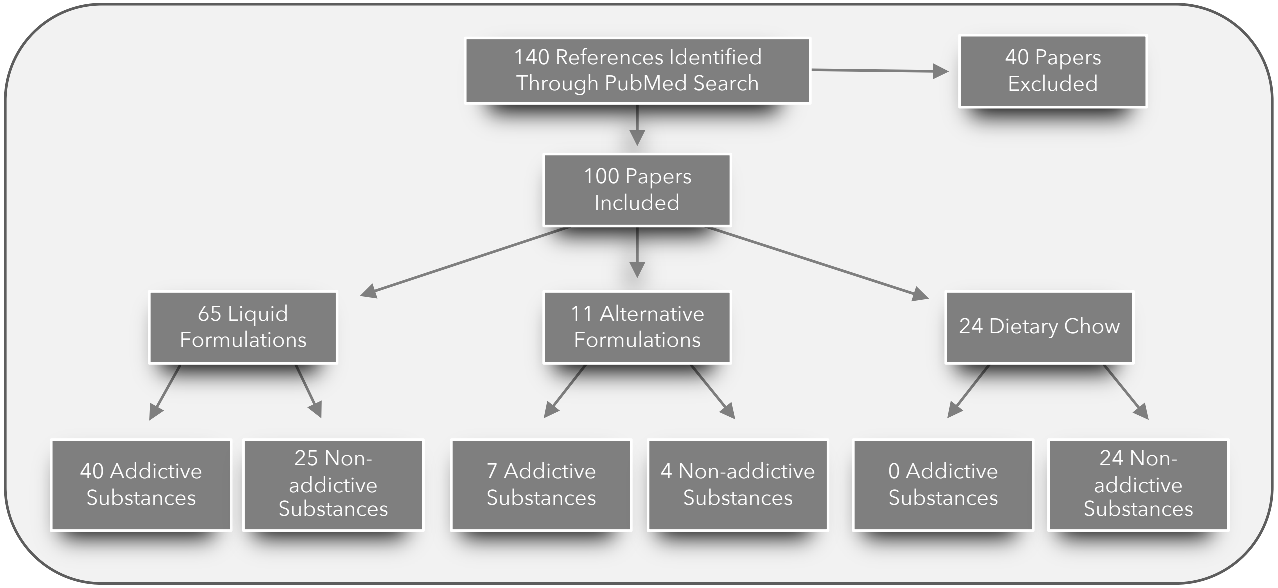

Supplemental Figure 1. A literature survey was completed on PubMed using the following search terms on October 31, 2025: "((((((dosing OR dosage OR dose) AND (animal OR rat OR mouse OR mice OR rats)) AND (voluntary)) AND (consumption)) AND (drug OR pharmaceutical OR compound))) NOT (alcohol)". Papers were categorized based on primary dosing method. Papers were excluded if they used oral gavage, injections, or infusions, or if they did not include an animal dosing method. Exclusion/inclusion criteria and further details are in **Supplemental Table 1**.
